# An Improved Unscented Kalman Filter Applied to Positioning and Navigation of Autonomous Underwater Vehicles

**DOI:** 10.3390/s25020551

**Published:** 2025-01-18

**Authors:** Jinchao Zhao, Ya Zhang, Shizhong Li, Jiaxuan Wang, Lingling Fang, Luoyin Ning, Jinghao Feng, Jianwu Zhang

**Affiliations:** College of Mechatronics Engineering, North University of China, Taiyuan 030051, China

**Keywords:** unscented Kalman filter, inertial navigation system, unmanned underwater vehicle, integrated navigation

## Abstract

To enhance the positioning accuracy of autonomous underwater vehicles (AUVs), a new adaptive filtering algorithm (RHAUKF) is proposed. The most widely used filtering algorithm is the traditional Unscented Kalman Filter or the Adaptive Robust UKF (ARUKF). Excessive noise interference may cause a decrease in filtering accuracy and is highly likely to result in divergence by means of the traditional Unscented Kalman Filter, resulting in an increase in uncertainty factors during submersible mission execution. An estimation model for system noise, the adaptive Unscented Kalman Filter (UKF) algorithm was derived in light of the maximum likelihood criterion and optimized by applying the rolling-horizon estimation method, using the Newton–Raphson algorithm for the maximum likelihood estimation of noise statistics, and it was verified by simulation experiments using the Lie group inertial navigation error model. The results indicate that, compared with the UKF algorithm and the ARUKF, the improved algorithm reduces attitude angle errors by 45%, speed errors by 44%, and three-dimensional position errors by 47%. It can better cope with complex underwater environments, effectively address the problems of low filtering accuracy and even divergence, and improve the stability of submersibles.

## 1. Introduction

Autonomous underwater vehicles (AUVs) integrating acoustic communication, intelligent control, energy storage, and multi-sensor technologies, are precocious unmanned underwater platforms possessing strong autonomy, superior concealment, and extensive operational ranges [1]. Their navigation systems typically rely on filtering algorithms for precise positioning. In view of the rapid attenuation of electromagnetic waves in water renders, conventional satellite navigation is unusable, which poses significant challenges to underwater navigation research [2].

Strapdown inertial navigation systems (SINSs) are generally employed for AUV autonomous navigation, offering advantages such as excellent stealth, high data rates, and comprehensive navigation parameters [3]. Nevertheless, SINS navigation errors accumulate over time. To alleviate error, auxiliary correction methods are typically adopted, mainly in two ways: first, periodic surfacing to receive satellite signals or other radio signals for position correction; second, utilization of underwater acoustic positioning technology to provide position information [4]. The Doppler Velocity Log (DVL)/SINS integrated navigation system is currently the most prevalent underwater AUV navigation system. The key factor in the integrated navigation approach is the fusion filtering algorithm. Numerous filtering methods have been proposed by researchers worldwide, with the Kalman filter receiving significant attention owing to its distinct advantages [5].

The Kalman filter has the characteristic of dealing with objects that are high-dimensional, nonstationary, and time-varying. As a recursive algorithm, it is particularly suitable for computer implementation. Therefore, since the Kalman filter was proposed, it has been widely used in the engineering field [6]. Kalman filtering is applicable to linear system models. In practical engineering applications, an integrated navigation system usually contains certain nonlinear characteristics. If Kalman filtering is directly used for filtering calculations, model approximation errors may be introduced [7]. Therefore, improved methods are constantly being evolved, among which the Extended Kalman Filter (EKF) and the Unscented Kalman Filter (UKF) are widely used. The EKF algorithm performs first-order linearization truncation on the Taylor expansion of the nonlinear state function and the measurement function and transforms the nonlinear filtering into a linear filtering problem. This algorithm is simple to calculate and has a fast convergence rate [8]. However, EKF has some shortcomings, such as the error caused by linearization truncation and the need to calculate the Jacobian matrix of the nonlinear function (the calculation load of the matrix in the computer increases significantly with the increase in the navigation filtering data dimension), which may lead to the inability of an unmanned vehicle to make timely control decisions when completing a task and even cause delays, resulting in task failure [9]. The UKF algorithm uses UT (Unscented Transformation) to approximate the posterior probability density of the nonlinear system and selects several points in the original state distribution according to certain rules so that the average covariance of each point is equal to the average covariance of the original state, so as to achieve the purpose of being fast and efficient [10]. This method does not require linearization of the function, nor does it need to ignore the high-order terms of the function. Therefore, the function obtained by this method has higher mean and covariance estimation accuracy [11].

Compared with the EKF, the UKF is simpler in terms of calculation and has high filtering accuracy. It has been widely used in the filtering solution of nonlinear equations. However, there are still problems of low filtering accuracy and even divergence [12]. Reference [13] proposed an asynchronous adaptive direct Kalman filter (AADKF) algorithm for an underwater integrated navigation system. The algorithm improves navigation accuracy by adaptively adjusting the measurement noise variance matrix and solves the problem of unknown measurement covariance matrices and their variation over time. Luo et al. [14] proposed a robust Kalman filter to eliminate process uncertainties and measurement anomalies caused by severe maneuvering, thereby improving navigation accuracy. Aiming at the large initial heading errors and horizontal attitude errors that may occur in AUVs, Lu Zhang et al. [15] proposed a new nonlinear attitude error model based on the square-root information filter (SR-CIF), which significantly improved the convergence rate of the initial alignment of a strapdown inertial navigation system. At present, research on the Kalman filter algorithm is still in progress. He Shan et al. [16] introduced an enhanced strong tracking quadrature Kalman filter algorithm. The incorporation of strong tracking filtering concepts into the nonlinear filtering framework resulted in improved filtering accuracy; however, issues of misjudgment and divergence persist. Ye Chen et al. [17] presented a strong tracking UKF algorithm. Through the refinement of the strong tracking filter, an algorithm with heightened accuracy and enhanced noise resistance was developed and applied to AUVs, yet there remains scope for efficiency enhancement. Chen Xiaofeng [18] proposed an integrated navigation method based on a strong tracking Unscented Kalman Filter algorithm. By combining the strong tracking algorithm with the Unscented Kalman Filter algorithm, the filtering accuracy error was reduced, but there is still a situation where the computational power is more complex. Chen Guangwu [19] proposed an integrated navigation algorithm based on an adaptive interacting multiple Kalman filter model. By calculating the residuals, the observation likelihood of the model is adaptively adjusted to improve the performance of state estimation. However, there are still some errors when the algorithm is applied underwater. In reference [20], a fault processing algorithm based on the Federated Kalman Filter (FKF), which combines the time update value of the fault sub-filter adaptively, was proposed. The FKF in the integrated navigation system was improved, reducing the pollution to other sub-filters caused by feedback and reducing abnormal positioning caused by faults. However, the noise processing ability is still weak, and the algorithm proposed in this paper has a better performance in noise processing, which can better increase the stability of submersibles and the accuracy of navigation. González et al. [21], in their research, combined the EKF with sonar data; the improved Kalman filter method significantly improved the navigation accuracy of underwater AUVs in deep-sea areas. However, when the statistical characteristics of the equivalent measurement noise change, the measurement rate and navigation accuracy decrease. These methods have the problems of insufficient computing power and insufficient accuracy in complex environments [22,23,24]. With the emergence of deep learning neural networks, the development of data-driven autonomous navigation control strategies has been overtaken. Methods of learning how to avoid collisions based on supervised deep learning path planning are becoming more and more popular. Wang et al. [25,26,27,28] designed the integral backstepping method to design a controller and realized the global trajectory tracking of mobile robots. The algorithm is simple and easy to transplant. Chen Ziyin et al. [29] proposed a backstepping control method based on feedback gain. By combining neural network control methods, the problem of depth control of autonomous underwater vehicles was solved. Gao [30] proposed an adaptive PD control algorithm, which has good stability and adaptability in following a preset trajectory. In particular, with the latest development of DL, the combination of deep continuous conditional random domains and deep convolutional neural networks is used to improve the accuracy of depth prediction [31]. These navigation methods indeed improve the navigation accuracy of unmanned underwater vehicles and are easy to combine and adjust. However, these methods also have certain defects, such as cumbersome control processes, complex environmental perception, and imprecise and updated system models. The algorithm proposed in this paper can reduce the impact of noise and other environmental conditions on underwater vehicles to improve the stability of submersibles [32,33].

In this paper, the Unscented Kalman Filter algorithm is optimized by introducing rolling-time-domain estimation, and the Newton–Raphon algorithm is used to solve the maximum likelihood estimation of noise statistics, aiming to reduce noise interference for submersibles and ensure that underwater vehicles can complete tasks smoothly in complex environments.

In view of the problems existing in the above methods, this paper proposes an adaptive filtering algorithm (RHAUKF) based on the maximum likelihood criterion and rolling-time-domain estimation. The main research work was as follows: (1) The Unscented Kalman Filter (UKF) was analyzed and an adaptive filtering algorithm (RHAUKF) was proposed. (2) A filtering scheme and simulation model suitable for the SINS/DVL integrated navigation system were designed, and the RHAUKF algorithm, the ARUKF algorithm, and the UKF algorithm were analyzed and compared with the model. Through the above research and comparison, it is hoped that the algorithm will better meet the working requirements of submersibles.

The remainder of this paper is structured as follows: Section 2 provides the basic form of the algorithm proposed in this paper. In Section 3, an adaptive UKF algorithm based on the maximum likelihood criterion and moving-horizon estimation is proposed, and the derivation process and implementation steps of the algorithm are briefly introduced. In Section 4, the correctness and feasibility of the three algorithms are verified, and through a comparison with the UKF algorithm and the ARUKF algorithm, the superiority of the proposed algorithm is highlighted. Section 5 is a summary of this article and an overview of the conclusions.

Table 1 presents the abbreviations, symbols and their corresponding meanings used in this paper; Table 2 summarizes the results of the literature review.

## 2. The Relevant Theory of Kalman Filtering

### 2.1. Unscented Kalman Filter (UKF)

The UKF is based on the Unscented Transform (UT), which uses a standard Kalman filter as the framework with deterministic sampling. Essentially, it is an approximate linear minimum-variance estimation method. Unlike the EKF, the UKF employs the UT to replace deterministic sampling. The UT is briefly described below.

#### 2.1.1. Unscented Transform (UT)

**Figure 1:**
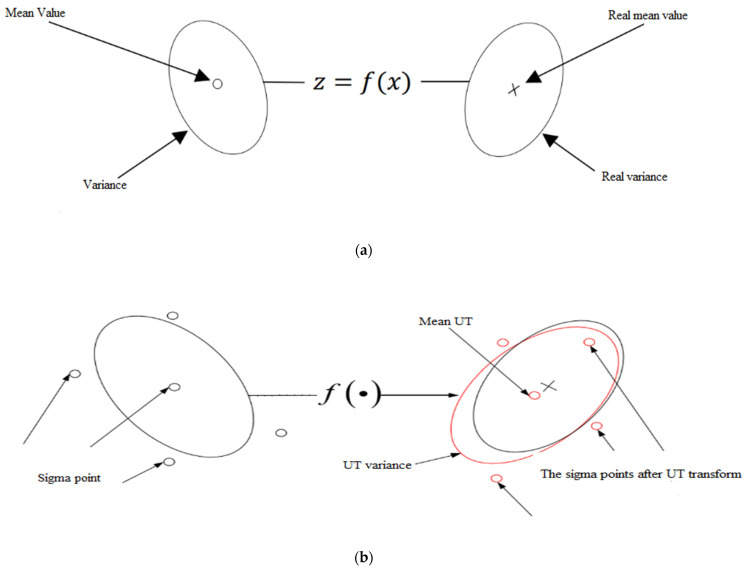
Illustration of the UT Transform: (**a**) actual; (**b**) UT Transform.

The UT first designs the sampling point set, $\left\{x_{i}\right\}$, and propagates its members through the function f(∙) to obtain a new set of points, $\left\{y_{i}\right\}$, and then calculates the posterior statistics $\left(y,P_{yy}\right)$ of the random variable. The UT process is illustrated in Figure 1, where the sample points are generally referred to as sigma points [26,27,28].

The Unscented Transform (UT) proceeds as follows:

1. Given an input variable (*x*) and its mean ($\bar{x}$) and covariance ($P_{xx}$), a sampling strategy is chosen to generate a set of sigma points ($\left\{x_{i}\right\},1,2,\ldots ,L$), along with corresponding weights ($W_{i}^{m}$ and $W_{i}^{c}$), where L represents the number of sigma points, $W_{i}^{m}$ is the weight for the mean, and $W_{i}^{c}$ is the weight for the covariance.
2. Each sigma point from the generated set ($\left\{x_{i}\right\},1,2,\ldots ,L$) is substituted into the nonlinear function (f(∙)) to obtain the transformed sigma point set ($\left\{y_{i}\right\}$), satisfying
  (1) $$y_{i}=f\left(x_{i}\right),i=1,\ldots ,L$$
3. The mean $\left(\bar{y}\right)$ and covariance ($P_{yy}$) of the output variable (y) are calculated by weighting sigma points $\left\{y_{i}\right\}$ in the above Equation (1). The weights of $\left\{y_{i}\right\}\;$ used here are the same weights ($W_{i}^{m}$ and $W_{i}^{c}$) assigned to the original sigma points ($\left\{x_{i}\right\},i=1,2,\ldots L$).

(2) $$\bar{y}=\overset{L-1}{\underset{i=0}{\sum }}W_{i}^{m}y_{i}$$

(3) $$P_{yy}=\overset{L-1}{\underset{i=0}{\sum }}W_{i}^{c}(y_{i}-\bar{y}){\left(y_{i}-\bar{y}\right)}^{T}$$

In the above calculation, if different sampling strategies are used, the sigma point set ($\left\{x_{i}\right\},1,2,\ldots ,L$) is generated. The forms of *L*, $W_{i}^{m}$, and $W_{i}^{c}$ are also different.

#### 2.1.2. The Unscented Kalman Filter (UKF) Algorithm

The Unscented Kalman Filter (UKF) approximates the probability density function of a nonlinear system to accurately transmit the mean and covariance of the state distribution without calculating the Jacobian matrix. It approximates the state distribution through a set of samples called sigma points. This deterministic sampling method requires fewer sigma points. The UT method improved by Julier et al. further reduces the number of sigma points and enhances the sampling accuracy. Overall, the UKF, as an improved Kalman filtering method, performs linear minimum-variance estimation through model transformation, effectively circumventing the linearization difficulties inherent in strongly nonlinear systems and resolving the linearization error issues present in the Extended Kalman Filter (EKF).

Assuming a nonlinear dynamic system in discrete time:

(4) $$\left\{\begin{array}{c} X_{k}=f\left(X_{k-1}\right)+W_{k-1}\\ Z_{k}=h\left(X_{k}\right)+V_{k} \end{array}\right.$$

In Equation (4), $X_{k}\in R^{n}$ and $Z_{k}\in R^{m}$ represent the system state vector and the measurement vector at time k, respectively; $W_{k}\in R^{n}$ and $V_{k}\in R^{m}$ represent the system state noise and the measurement noise, respectively; f(∙) and h(∙) are the nonlinear system state function and the measurement function, respectively; and $W_{k}$ and $V_{k}$ are uncorrelated Gaussian white noise processes, whose statistical properties satisfy the rule of a normal distribution. These sample points, after transformation through the nonlinear system, generate corresponding sigma points. Subsequently, further calculations based on these transformed points yield the mean, covariance, and other statistical characteristics of the state.

For Equations (4) and (5), the UKF filtering algorithm steps are as follows:

1. Initialization
  (5) $$\left\{\begin{array}{c} {\hat{X}}_{0}=E\left[X_{0}\right]\\ P_{0}=\left[\left(X_{0}-{\hat{X}}_{0}\right){\left(X_{0}-{\hat{X}}_{0}\right)}^{T}\right] \end{array}\right.$$
2. Sigma Point Sampling and Time Update
  (6) $$\left\{\begin{array}{l} X_{0,k-1}={\hat{X}}_{k-1}\\ X_{i,k-1}={\hat{X}}_{k-1}+{\left(\sqrt{\left(n+\lambda \right)P_{k-1}}\right)}_{i},i=1,2,\ldots ,n\\ X_{i,k-1}={\hat{X}}_{k-1}-{\left(\sqrt{\left(n+\lambda \right)P_{k-1}}\right)}_{i-n},i=n+1,n+2,\ldots ,2n \end{array}\right.$$
  (7) $$X_{i,k/k-1}^{*}=f(X_{i,k/k-1})+q_{k},i=0,1,2,\ldots ,2n$$
  (8) $${\hat{X}}_{k/k-1}=\sum_{i=0}^{2n}W_{i}^{m}X_{i,k/k-1}^{*}=\sum_{i=0}^{2n}W_{i}^{m}f(X_{i,k-1})+q_{k}$$
  (9) $$P_{k/k-1}=\overset{2n}{\underset{i=0}{\sum }}W_{i}^{c}\left(X_{i,k/k-1}^{*}-{\hat{X}}_{k/k-1}\right){\left(X_{i,k/k-1}^{*}-{\hat{X}}_{k/k-1}\right)}^{T}+Q_{k}$$
  (10) $$\left\{\begin{array}{l} X_{0,k/k-1}={\hat{X}}_{k/k-1}\\ X_{i,k/k-1}={\hat{X}}_{k|k-1}+{\left(\sqrt{\left(n+\lambda \right)P_{k/k-1}}\right)}_{i},i=1,2,\ldots ,n\\ X_{i,k/k-1}={\hat{X}}_{k/k-1}-{\left(\sqrt{\left(n+\lambda \right)P_{k/k-1}}\right)}_{i-n},i=n+1,n+2,\ldots ,2n \end{array}\right.$$
  (11) $$\gamma_{i,k/k-1}=h(X_{i,k/k-1})+r_{k}$$
  (12) $${\hat{Z}}_{k/k-1}=\sum_{i=0}^{2n}W_{i}^{m}\gamma_{i,k/k-1}=\overset{2n}{\underset{i=0}{\sum }}W_{i}^{m}h\left(X_{i,k/k-1}\right)+r_{k}$$

The weights corresponding to the sigma points are as follows:

(13) $$\left\{\begin{array}{l} W_{0}^{m}=\frac{\lambda }{n+\lambda }\\ W_{0}^{c}=\frac{\lambda }{n+\lambda }+(1-\alpha^{2}+\beta )\\ W_{i}^{m}=W_{i}^{c}=\frac{1}{2(n+\lambda )},i=1,2,\ldots ,2n\\ \lambda =\alpha^{2}(n+k)-n \end{array}\right.$$

where ${\left(\sqrt{\left(n+\lambda \right)P_{k-1}}\right)}_{i}$ is the ith column of the triangular decomposition square root of $(n+\lambda )P_{k-1}$ and α is the adjustment parameter. It is advisable to control the distribution of sigma points around ${\hat{X}}_{k-1}$. The range can be taken as $10^{-4}\leq \alpha \leq 1;\;k=3-n$.

3. Measurement Update
  (14) $$P_{{\hat{Z}}_{k/k-1}}=\overset{2n}{\underset{i=0}{\sum }}W_{i}^{c}\left(\gamma_{i,k/k-1}-{\hat{Z}}_{k/k-1}\right){\left(\gamma_{i,k/k-1}-{\hat{Z}}_{k/k-1}\right)}^{T}+R_{k}$$
  (15) $$P_{{\hat{X}}_{k/k-1}-{\hat{Z}}_{k/k-1}}=\sum_{i=0}^{2n}W_{i}^{c}(X_{i,k/k-1}^{*}-{\hat{X}}_{k/k-1}){\left(\gamma_{i_{i},k/k-1}-{\hat{Z}}_{k/k-1}\right)}^{T}$$
  (16) $$K_{k}=P_{{\hat{X}}_{k/k-1}{\hat{Z}}_{k/k-1}}P_{{\hat{Z}}_{k/k-1}}^{-1}$$
  (17) $${\hat{X}}_{k}={\hat{X}}_{k/k-1}+K_{k}(Z_{k}-Z_{k/k-1})$$
  (18) $$P_{k}=P_{k,k-1}-K_{k}P_{{\hat{Z}}_{k/k-1}}K_{k}^{T}$$

## 3. An Adaptive UKF Algorithm Based on Maximum Likelihood Estimation and Sliding-Window Estimation

The Unscented Kalman Filter (UKF) algorithm is sensitive to initial values. If there is a large deviation in the initial values or the system model is subject to abnormal interference, this may lead to a decrease in filtering accuracy. Furthermore, the prior statistical characteristics of the system noise must be accurate or known to improve the filtering performance. In reference [24], an adaptive robust UKF (Adaptive Robust UKF, ARUKF) was proposed. This algorithm combines the UKF with robust adaptive technology. By adopting the concept of Marginalized Unscented Transformation (MUT) according to the characteristics of the combination model, introducing an adaptive factor to adjust the weights of UKF observation data and prediction data effectively suppresses the abnormal interference in the system model. However, when the statistical characteristics of the measurement noise change, the advantage of this method is no longer so evident.

Adaptive filtering algorithms incorporating maximum likelihood estimation (MLE) can automatically adjust filter coefficients based on the statistical relationship between the input and the expected output signals to optimize filter performance. However, these algorithms are computationally intensive, requiring iterative parameter updates, and their performance degrades when signal statistics change or when accurate desired output estimation is difficult. This is particularly problematic in nonlinear systems, where real-time performance suffers, limiting practical applications. To address these challenges, a Recursive Hybrid Adaptive UKF (RHAUKF) algorithm based on MLE and a sliding-window estimation approach has been proposed. This algorithm optimizes noise statistical characteristics using sliding-window estimation and employs the Newton–Raphson method for noise estimation, thereby improving UKF stability and accuracy in underwater navigation applications. 

### 3.1. Noise Statistical Estimation Model Based on the Maximum Likelihood Criterion

Firstly, the estimation model of system noise statistics was constructed according to the maximum likelihood criterion. Assuming that the statistical characteristics of the system noise to be estimated are $\theta =\left\{q,Q,r,R\right\}$, the system parameter estimation based on the maximum likelihood criterion is as follows:

(19) $$\hat{\theta }=\arg\underset{\theta }{\max}\left\{\ln\left[L\left(\theta |Z_{1:k}\right)\right]\right\}$$

In Equation (12), $\ln\left[L(\theta |Z_{1:k})\right]$ is the log-likelihood function of the parameter θ.

According to the definition of the likelihood function, the following formula can be obtained:

(20) $$L\left(\theta |Z_{1:k}\right)=p\left(Z_{1:k}|\theta \right)$$

In the above formula, $p\left(Z_{1:k}|\theta \right)$ represents the joint probability density function of $Z_{1},Z_{2},\ldots ,Z_{k}$.

If the prediction residual (innovation) vector ($\varepsilon_{k}$) of the system satisfies the following:

(21) $$\varepsilon_{k}=Z_{k}-{\hat{Z}}_{k/k-1}=Z_{k}-\sum_{i=0}^{2n}\omega_{i}^{m}h\left(X_{i,k/k-1}\right)-r_{k}$$

there is zero-mean Gaussian white noise, i.e., $\varepsilon_{k}\sim N(0,\sum_{K})$, where

(22) $$\Sigma_{k}=E\left[\varepsilon_{k}\varepsilon_{k}^{T}\right]=\sum_{i=0}^{2n}\omega_{i}^{c}\left(\Upsilon_{i,k/k-1}-{\hat{Z}}_{k/k-1}\right){\left(\Upsilon_{i,k/k-1}-{\hat{Z}}_{k/k-1}\right)}^{T}+R_{k}$$

The likelihood function of the parameter θ represented by the innovation vector is as follows:

(23) $$L\left(\theta |Z_{1:k}\right)=p\left(Z_{1:k}|\theta \right)\approx \prod_{i=1}^{k}p\left(\varepsilon_{i}|\theta \right)=\prod_{i=1}^{k}\frac{1}{{\left(2\pi \right)}^{\frac{n}{2}}{\left|\Sigma_{i}\right|}^{\frac{1}{2}}}\exp\left\{-\frac{\varepsilon_{i}^{T}\Sigma_{i}^{-1}\varepsilon_{i}}{2}\right\}$$

Ignoring the constant term, the log-likelihood function of the parameter θ can be expressed as follows:

(24) $$\ln\left[L\left(\theta |Z_{1:k}\right)\right]\approx -\frac{1}{2}\sum_{i=1}^{k}\left(\ln\left|\Sigma_{i}\right|+\varepsilon_{i}^{T}\Sigma_{i}^{-1}\varepsilon_{i}\right)$$

Then, the noise statistical estimation based on the maximum likelihood criterion is as follows:

(25) $$\left\{\begin{array}{l} {\hat{\theta }}_{ML}=\arg\underset{\theta }{\min}\left[\sum_{i=1}^{k}\left(\ln\left|\Sigma_{i}\right|+\varepsilon_{i}^{T}\Sigma_{i}^{-1}\varepsilon_{i}\right)\right]\\ Q>0\\ R>0 \end{array}\right.$$

The matrices Q and R are positive definite matrices, assuming that they are diagonal matrices, namely:

(26) $$\begin{array}{l} Q=diag\left[a_{1}a_{2}\cdots a_{n}\right]\\ R=diag\left[b_{1}b_{2}\cdots b_{m}\right] \end{array}$$

At this time, the parameter estimation of $\theta =\left\{q,Q,r,R\right\}$ based on the maximum likelihood criterion is as follows:

(27) $$\left\{\begin{array}{l} {\hat{\theta }}_{ML}=\arg\underset{\theta }{\min}\left[\sum_{i=1}^{k}\left(\ln\Sigma_{i}\right)+\varepsilon_{i}^{T}\Sigma_{i}^{-1}\varepsilon_{i}\right]\\ Q=diag\left[a_{1}a_{2}\cdot \cdot \cdot a_{n}\right]\\ R=diag\left[b_{1}b_{2}\cdot \cdot \cdot b_{m}\right]\\ Q>0\\ R>0 \end{array}\right.$$

For the above formula, the estimated value of the time, k, can be solved by the Newton–Raphson algorithm, $\theta_{k}=\left\{q_{k},Q_{k},r_{k},R_{k}\right\}$, according to the algorithm ${\hat{\theta }}_{k}=\left\{{\hat{q}}_{k},{\hat{Q}}_{k},{\hat{r}}_{k},{\hat{R}}_{k}\right\}$.

### 3.2. Optimization of Noise Statistical Estimation Model Based on Moving-Horizon Estimation

For the noise statistics based on the maximum likelihood criterion, with the increase in time, k, the measurement and the innovation vector increase. With the increase in the measurement and innovation vectors, the maximum likelihood estimations of noise statistics will diverge, leading to filtering divergence. In order to solve the above problems, the concept of receding-horizon estimation (RHE) was introduced to optimize the above model and reduce the complexity of the model. The rolling-horizon estimation can optimize the model, and the process is as follows:

(28) $$\begin{array}{l} \underset{\theta }{\min}\phi (k,\theta )\\ \phi (k,\theta )=\sum_{i=0}^{k}(\ln\left|\Sigma_{i}\right|+\varepsilon_{i}^{T}\Sigma_{i}^{-1}\varepsilon_{i}) \end{array}$$

The following approximate moving-horizon estimation problem is used to replace the optimization problem in the above formula.

(29) $$\begin{array}{cc} & \underset{\theta }{\min}J(N,\theta )\\ J(N,\theta ) & =\sum_{i=k-N+1}^{k}\left(\ln\left|\Sigma_{i}\right|+\varepsilon_{i}^{T}\Sigma_{i}^{-1}\varepsilon_{i}\right)+\sum_{i=1}^{k-N}\left(\ln\left|\Sigma_{i}\right|+\varepsilon_{i}^{T}\Sigma_{i}^{-1}\varepsilon_{i}\right)\\ & =\sum_{i=k-N+1}^{k}\left(\ln\left|\Sigma_{i}\right|+\varepsilon_{i}^{T}\Sigma_{i}^{-1}\varepsilon_{i}\right)+\phi (k-N,\theta ) \end{array}$$

Using the minimum value $\phi^{*}(k-N,\theta )$ of the objective function ϕ(k−N,θ), at the time k−N, instead of ϕ(k−N,θ) in the optimization problem (29), the optimization problem 3–30 is equivalent to the following:

(30) $$\begin{array}{l} \underset{\theta }{\min}J(N,\theta )\\ J(N,\theta )=\sum_{i=k-N+1}^{k}\left(\ln\left|\Sigma_{i}\right|+\varepsilon_{i}^{T}\Sigma_{i}^{-1}\varepsilon_{i}\right) \end{array}$$

Combined with Formula (27), the optimization problem can be expressed as follows:

(31) $$\left\{\begin{array}{l} {\hat{\theta }}^{*}=\arg\underset{\theta }{\min}\left[\sum_{i=k-N+1}^{k}\left(\ln\left|\Sigma_{i}\right|+\varepsilon_{i}^{T}\Sigma_{i}^{-1}\varepsilon_{i}\right)\right]\\ Q=diag\left[a_{1}a_{2}\cdots a_{n}\right]\\ R=diag\left[b_{1}b_{2}\cdots b_{n}\right]\\ Q>0\\ R>0 \end{array}\right.$$

### 3.3. Implementation and Evaluation of an Adaptive Algorithm

The discrete-time nonlinear system model is given below:

(32) $$\left\{\begin{array}{c} X_{k}=f\left(X_{k-1}\right)+W_{k}\\ Z_{k}=H_{k}X_{k}+V_{k} \end{array}\right.$$

In Equation (10), $H_{k}$ represents the measurement matrix; other variables are defined as in Equation (4). For nonlinear systems, the prediction residual (innovation) vector is defined as follows:

(33) $$\varepsilon_{k}=Z_{k}-H_{k}{\hat{X}}_{k/k-1}-r_{k}$$

Therefore,

(34) $$\left\{\begin{array}{c} E\left[\varepsilon_{k}]=E[Z_{k}-H_{k}{\hat{X}}_{k/k-1}-r_{k}\right]=0\\ \sum k=E[\varepsilon_{k}\varepsilon_{k}^{T}]=E[\left(Z_{k}-H_{k}{\hat{X}}_{k/k-1}-r_{k}\right)\left(Z_{k}-H_{k}{\hat{X}}_{k/k-1}-r_{k}{)}^{T}\right]=H_{k}P_{k/k-1}H_{k}^{T}+R_{k} \end{array}\right.$$

The RHAUKF algorithm proceeds as follows:

1. Initialization
  (35) $$\left\{\begin{array}{c} {\hat{X}}_{0}=E\left[X_{0}\right]\\ P_{0}=E\left[\left(X_{0}-{\hat{X}}_{0}\right){\left(X_{0}-{\hat{X}}_{0}\right)}^{T}\right] \end{array}\right.$$
2. Noise Estimation

The prediction residual (innovation) vector, $\varepsilon_{k}$, and its covariance matrices, ${\hat{X}}_{k-1}$ and $P_{k-1}$, at the moment, k, are obtained by simultaneous equations 3–7~3–14 and 3–22~3–23, and the optimization problem 3–32 is constructed. The estimated value of noise statistical characteristics at the moment is obtained by the Newton–Raphson algorithm, ${\hat{\theta }}_{k}^{*}=\left\{{\hat{q}}_{k}^{*},{\hat{Q}}_{k}^{*},{\hat{r}}_{k}^{*},{\hat{R}}_{k}^{*}\right\}$.

3. Time update

According to Formulas (6)~(12) and step (2), the equation ${\hat{\theta }}_{k}^{*}=\left\{{\hat{q}}_{k}^{*},{\hat{Q}}_{k}^{*},{\hat{r}}_{k}^{*},{\hat{R}}_{k}^{*}\right\}$ is obtained. The one-step prediction of the system’s state is calculated as follows:

(36) $${\hat{X}}_{k/k-1}=\sum_{i=0}^{2n}\omega_{i}^{m}\chi_{i,k/k-1}^{*}=\sum_{i=0}^{2n}\omega_{i}^{m}f\left(\chi_{i,k-1}\right)+{\hat{q}}_{k}^{*}$$

The one-step prediction error covariance matrix is calculated as follows:

(37) $$P_{k/k-1}=\sum_{i=0}^{2n}\omega_{i}^{c}\left(\chi_{i,k/k-1}^{*}-{\hat{X}}_{k/k-1}\right){\left(\chi_{i,k/k-1}^{*}-{\hat{X}}_{k/k-1}\right)}^{T}+{\hat{Q}}_{k}^{*}$$

And one-step prediction is measured by the following algorithm:

(38) $${\hat{Z}}_{k/k-1}=\sum_{i=0}^{2n}\omega_{i}^{m}\gamma_{i,k/k-1}=\sum_{i=0}^{2n}\omega_{i}^{m}h\left(\chi_{i,k/k-1}\right)+{\hat{r}}_{k}^{*}$$

4. Measurement Update

According to Formulas (14)~(18), ${\hat{X}}_{k}$ and $P_{k}$ are calculated, where

(39) $$P_{{\hat{Z}}_{k/k-1}}=\sum_{i=0}^{2n}\omega_{i}^{c}\left(\gamma_{i,k/k-1}-{\hat{Z}}_{k/k-1}\right){\left(\gamma_{i,k/k-1}-{\hat{Z}}_{k/k-1}\right)}^{T}+{\hat{R}}_{k}^{*}$$

**Figure 2:**
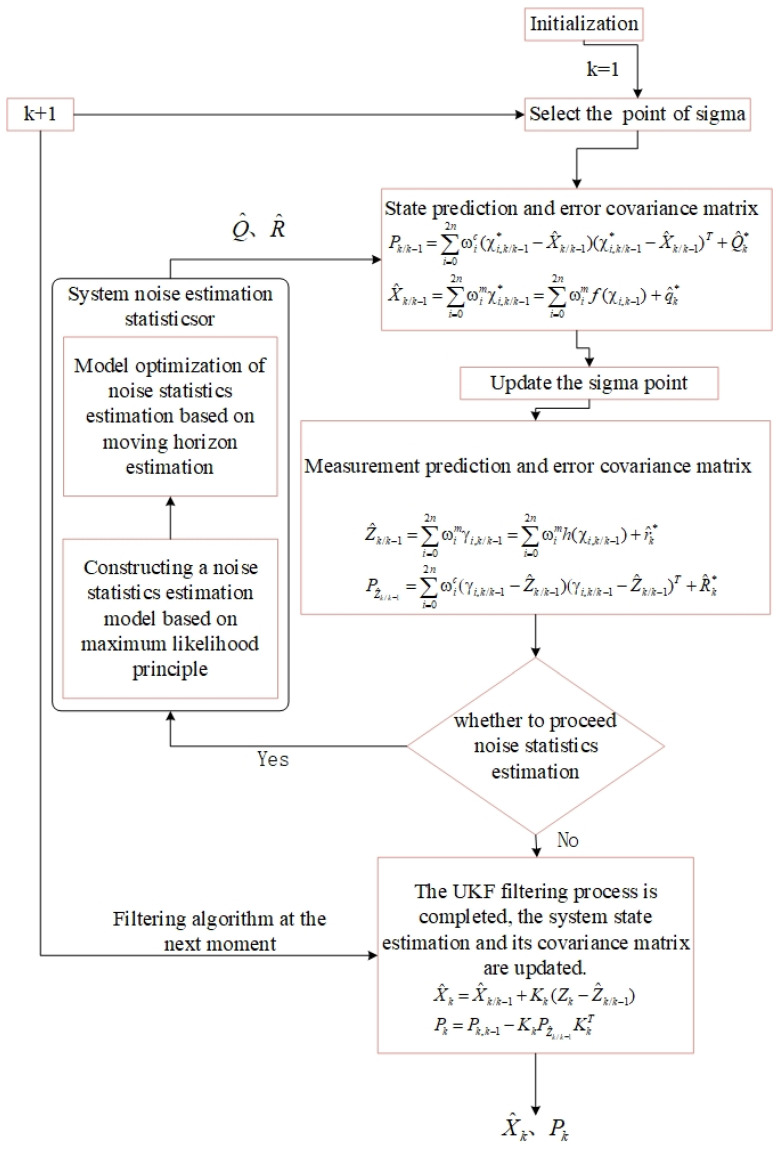
Flowchart of the RHAUKF algorithm.

The algorithm flowchart is shown in Figure 2.

## 4. Simulation Experiments and Analysis

This section presents simulation results comparing the performance of the UKF, ARUKF, and RHAUKF algorithms under noisy conditions. The Lie-group-based strapdown inertial navigation error model was employed. The trajectory of an underwater vehicle was simulated using MATLAB 2022 software, based on typical vehicle motion profiles. The results were analyzed and compared using the root mean square error (RMSE) and bias (BIAS). The root mean square error (RMSE), calculated using Equation (40), served as the performance metric to evaluate the accuracy and stability of the prediction models. A lower RMSE indicates a smaller difference between predicted and actually observed values, signifying higher prediction accuracy. Conversely, a higher RMSE indicates greater deviation and lower accuracy. BIAS refers to the systematic difference between the estimated value and the real parameter value. Using Formula (41) for calculation and comparison, a higher BIAS value shows that the model is too simple to capture the complex distribution of data, resulting in underfitting, and vice versa.

(40) $$RMSE\left(v\right)=\sqrt{\overset{M}{\underset{n=1}{\sum }}{\left|\left|v^{n}-v^{0}\right|\right|}^{2}/M}$$

(41) $$Bias=E\left[\hat{f}(x)\right]-f\left(x\right)$$

where $V^{n}$ is the estimated value of $v^{0}$ in the nth simulation experiment, M is the time period of the simulation experiment, $E\left[\cdot \right]$ denotes the sign of the expectation (i.e., the mean), $\hat{f}\left(x\right)$ denotes the model’s prediction of a given input, and $f\left(x\right)$ denotes the real function value.

**Figure 3:**
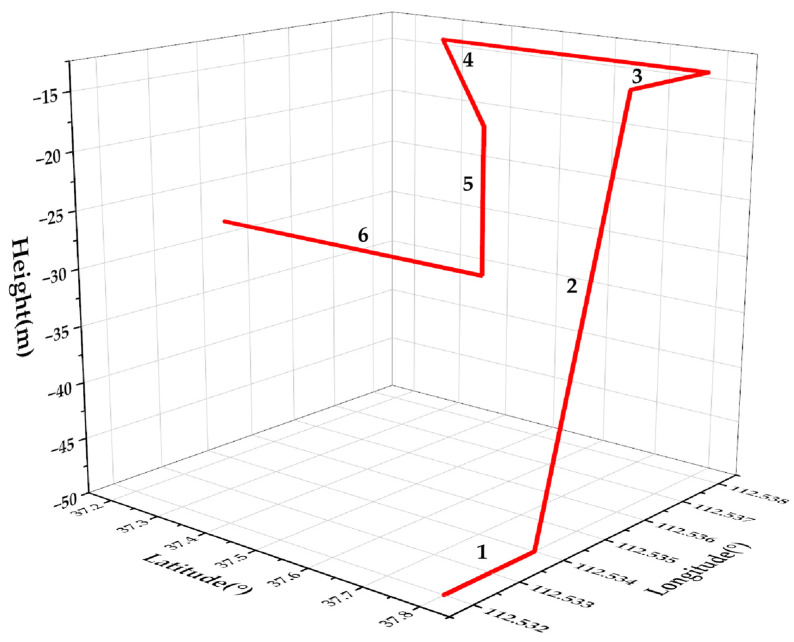
Underwater vehicle trajectories.

Figure 3 illustrates the simulated trajectories. Trajectories 1 and 6 represent constant horizontal velocities, trajectory 2 represents an ascent, trajectories 3 and 4 represent turns, and trajectory 5 represents a descent. These trajectories represent typical underwater vehicle maneuvers relevant to underwater navigation applications.

According to the trajectory profiles described above, the proposed RHAUKF algorithm was implemented within a Lie group-based strapdown inertial navigation error model. Its performance was compared with that of the standard UKF and the ARUKF algorithms previously discussed, all within the context of underwater navigation.

The initial position of the underwater vehicle (UUV) was set at a longitude (L) = 112.532° E, a latitude (λ) = 37.8° N, and a depth = 50 m, with a target final position error of 0. The Earth’s rotation rate is $\omega_{ie}=7.29\times 10^{-5}\;rad/s$; the variation of gravity (g) with latitude was neglected, and g was set to 9.78 m/s^2^. The initial UUV velocities were 5 m/s eastward, 0 m/s northward, and 0 m/s downward, with initial velocity errors of 0.1 m/s in all three directions. For the simulation, constant gyro drifts of 0.1°/h and random drifts of 0.01°/h were assumed along all three axes (east, north, and down). The accelerometer bias was set to $10^{-3}$ g, with a random drift of $10^{-4}\;g\sqrt{s}$.

The initial alignment error of the strapdown inertial navigation system was 0, and the initial attitude (heading angle, pitch angle, and roll angle) errors of the carrier were selected as (1°, 1°, 1°), (0.7°, 0.7°, 0.7°), and (0.5°, 0.5°, 0.5°); the initial position errors (longitude, latitude, and altitude) were set to (12 m, 12 m, 12 m). The Doppler Velocity Log (DVL) had a velocity measurement error of 0.3 m/s and a scale factor error of 0.1. The SINS sampling period was 0.01 s, the DVL sampling period was 0.2 s, the filter update cycle was 1 s, and the sliding-window length for the recursive estimation was 20 samples. With (1°, 1°, 1°) as the first case, (0.7°, 0.7°, 0.7°) as the second case, and (0.5°, 0.5°, 0.5°) as the third case, the other variables did not change.

The simulation time was selected as 5000 s to verify the performance of the algorithm. The time periods during which the process noise variance changed were as follows: $T_{1}$, from 2000 s to 2500 s, and $T_{2}$, from 3500 s to 4000 s. To better verify the filtering performance of the proposed algorithm when system noise statistics are unknown, it was assumed that during $T_{1}$ the covariance matrix of the process noise suddenly increased to four times the true value; during $T_{2}$, the covariance matrix of the measurement noise suddenly increased to five times the true value.

**Figure 4:**
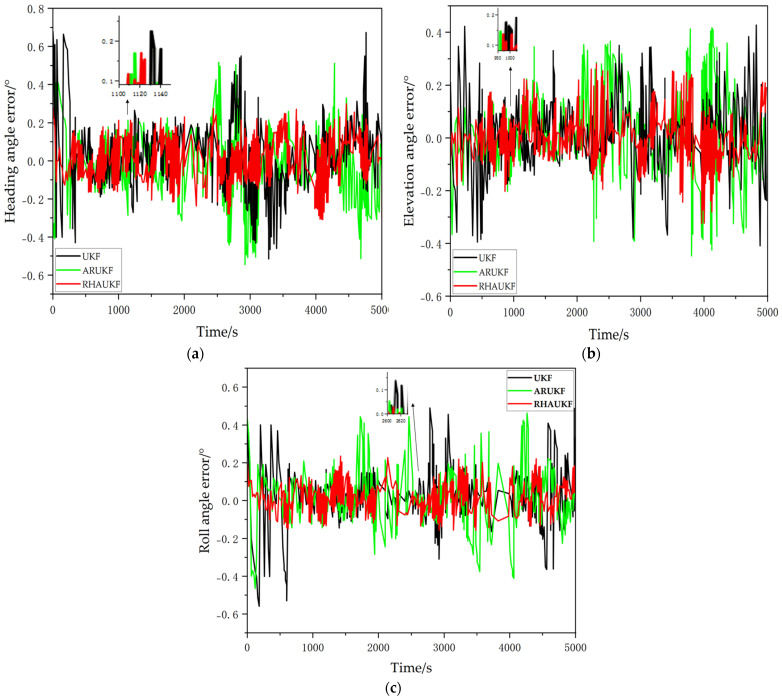
In the first case, the attitude angle error curves under the three algorithms: (**a**) heading angle errors; (**b**) pitch angle errors; (**c**) roll angle errors.

The simulation results are shown in Figure 4.

The heading, pitch, and roll error curves obtained using the three different filtering algorithms are shown in Figure 4. When accurate noise statistics are available, the traditional UKF, the ARUKF, and the proposed RHAUKF exhibit comparable accuracy in estimating heading, pitch, and roll, with errors generally within the ranges of [−0.25°, 0.28°], [−0.20°, 0.20°], and [−0.18°, 0.20°], respectively, demonstrating their effectiveness in typical underwater navigation scenarios.

During periods $T_{1}$ and $T_{2}$, the increased measurement noise covariance resulted in degraded heading, pitch, and roll estimation accuracy for the standard UKF. Specifically, the UKF exhibited heading, pitch, and roll errors within the ranges of [−0.55°, 0.58°], [−0.48°, 0.45°], and [−0.40°, 0.60°] during $T_{1}$ and within the ranges of [−0.68°, 0.80°], [−0.70°, 0.55°], and [−0.73°, 0.71°] during $T_{2}$. While the ARUKF showed improved accuracy compared to the UKF, its errors remained relatively large under the increased noise conditions, specifically within the ranges of [−0.48°, 0.50°], [−0.38°, 0.33°], and [−0.30°, 0.50°] during $T_{1}$ and within the ranges of [−0.58°, 0.60°], [−0.51°, 0.43°], and [−0.32°, 0.58°] during $T_{2}$. In contrast, the proposed RHAUKF algorithm maintained significantly higher accuracy, with errors remaining within the ranges of [−0.26°, 0.28°], [−0.22°, 0.30°], and [−0.18°, 0.28°] during $T_{1}$ and within the ranges of [−0.23°, 0.28°], [−0.25°, 0.23°], and [−0.25°, 0.30°] during $T_{2}$, demonstrating a substantial improvement in filtering accuracy and robustness against uncertain process noise, which is a critical advantage for reliable underwater navigation.

**Figure 5:**
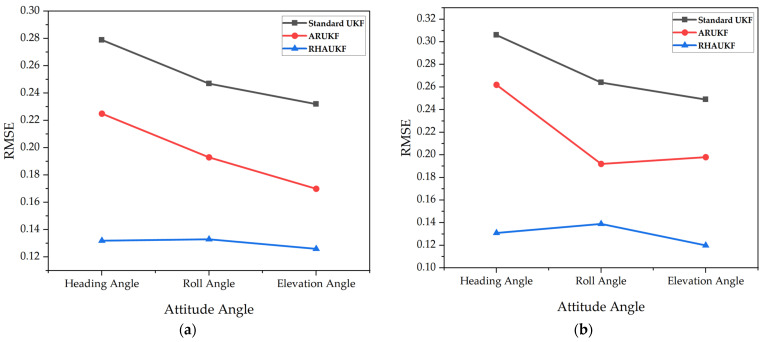
The root mean square errors of the attitude angle parameters: (**a**) the errors in the time period *T*_1_; (**b**) the errors in the time period *T*_2_.

**Figure 6:**
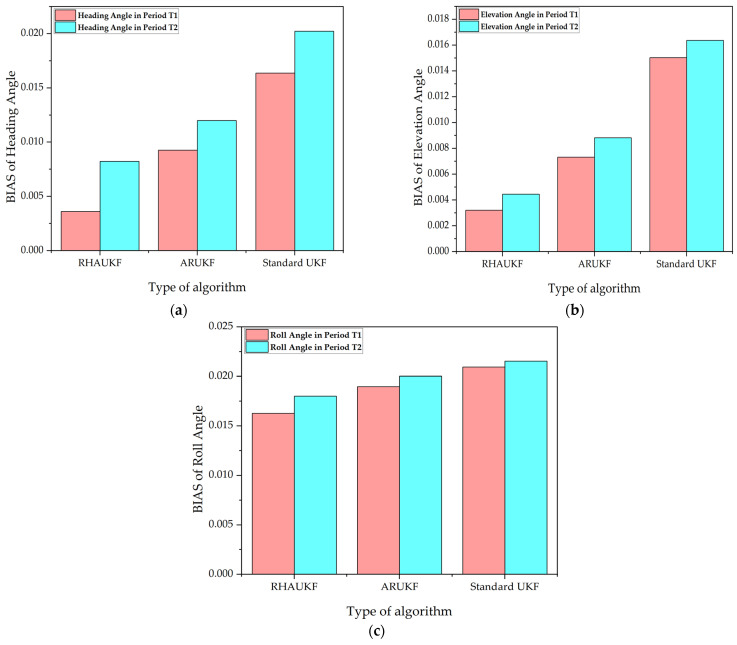
Deviations of attitude angle parameters: (**a**) BIAS of heading angle; (**b**) BIAS of elevation angle; (**c**) BIAS of roll angle.

Figure 5 shows the root mean square error of the attitude angle parameters obtained by the three algorithms in one case under $T_{1}$ and $T_{2}$. Figure 6 shows deviation diagrams of the attitude angle parameters obtained.

From Figure 5, the root mean square error of the attitude angle of the RHAUKF was the smallest during the two time periods, $T_{1}$ and $T_{2}$, and it was significantly smaller than that of the classical UKF and the ARUKF. Figure 6 indicates that the deviation of the RHAUKF was the smallest during the two time periods, $T_{1}$ and $T_{2}$, and it was significantly smaller than those of the classical UKF and the ARUKF.

**Figure 7:**
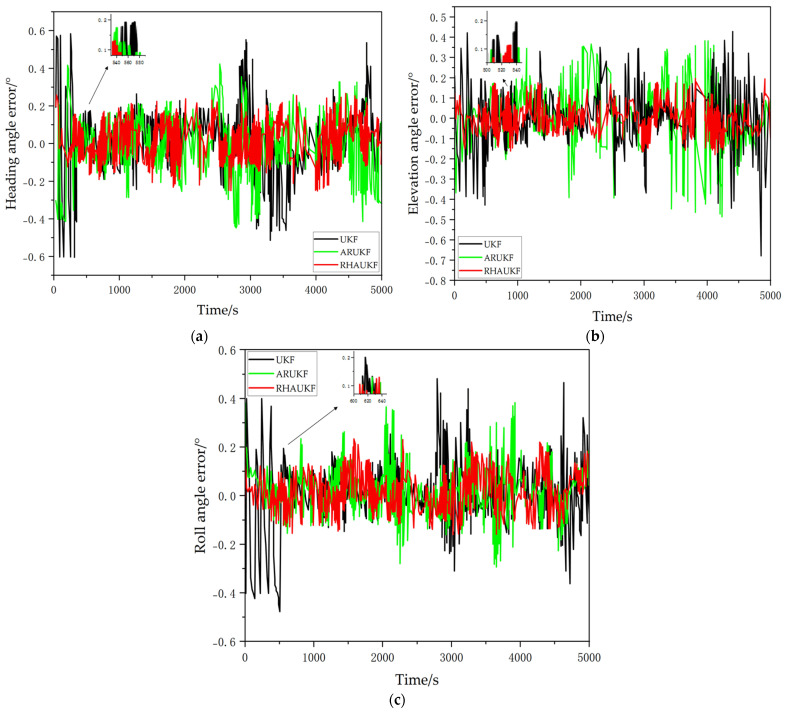
The attitude angle error curves under the three algorithms in the second case: (**a**) heading angle errors; (**b**) pitch angle errors; (**c**) roll angle errors.

Figure 7 reveals the error curves for the heading angle, pitch angle, and roll angle calculated by the three different filtering algorithms in the second case. When accurate noise statistical characteristics were obtained, the traditional UKF algorithm, the ARUKF algorithm, and the proposed RHAUKF algorithm achieved similar estimation accuracies for the heading angle, pitch angle, and roll angle, and the errors were approximately within the ranges of [−0.21°, 0.26°], [−0.19°, 0.18°], and [−0.15°, 0.17°], respectively.

At $T_{1}$ and $T_{2}$, due to the covariance matrices of the measurement noise becoming larger, the estimation accuracy of the UKF for heading angle, pitch angle, and roll angle was reduced. The heading angle, pitch angle, and roll angle errors obtained by the UKF in the two time periods were [−0.49°, 0.54°], [−0.44°, 0.39°], and [−0.35°, 0.52°] and [−0.61°, 0.74°], [−0.67°, 0.51°], and [−0.68°, 0.66°], respectively. The heading angle, pitch angle, and roll angle errors obtained by the ARUKF in these two time periods were [−0.42°, 0.47°], [−0.34°, 0.28°], and [−0.26°, 0.43°] and [−0.54°, 0.52°], [−0.45°, 0.35°], and [−0.27°, 0.52°], respectively. Although the accuracy was improved compared with the UKF, the error under this algorithm was still large. The errors of the proposed RHAUKF algorithm in these two time periods were [−0.23°, 0.22°], [−0.18°, 0.23°], and [−0.15°, 0.23°] and [−0.17°, 0.24°], [−0.23°, 0.18°], and [−0.19°, 0.23°], respectively. Compared with the UKF and the ARUKF, the accuracies for heading angle, pitch angle, and roll angle were the highest, and the filtering accuracy was greatly improved, which effectively suppressed the interference of uncertain process noise.

As shown in Figure 7, when the statistical characteristics of the noise were accurately known, the estimation accuracies of the three methods for the three-way velocity were equivalent, and the obtained east, north, and sky velocity errors were about [−0.11 m/s, 0.13 m/s], [−0.12 m/s, 0.10 m/s], and [−0.15 m/s, 0.17 m/s].

In the time period when the noise variance changed in the two processes of $T_{1}$ and $T_{2}$, the east, north, and sky velocity errors obtained by the UKF were about [−0.25 m/s, 0.28 m/s], [−0.29 m/s, 0.31 m/s], and [−0.28 m/s, 0.33 m/s] and [−0.35 m/s, 0.35 m/s], [−0.36 m/s, 0.38 m/s], and [−0.37 m/s, 0.38 m/s], respectively. The three-way velocity errors obtained by the ARUKF in these two periods were [−0.23 m/s, 0.18 m/s ], [−0.19 m/s, 0.19 m/s], and [−0.26 m/s, 0.22 m/s] and [−0.18 m/s, 0.26 m/s], [−0.20 m/s, 0.19 m/s], and [−0.25 m/s, 0.30 m/s], respectively. The accuracy of the improved algorithm is limited. The proposed RHAUKF algorithm performs best in terms of three-way velocity error accuracy. In the two time periods, the velocity errors in the east, north, and sky directions were the most accurate when the noise statistical characteristics were known, and the filtering accuracy was significantly better than that of the UKF and the ARUKF algorithms.

**Figure 8:**
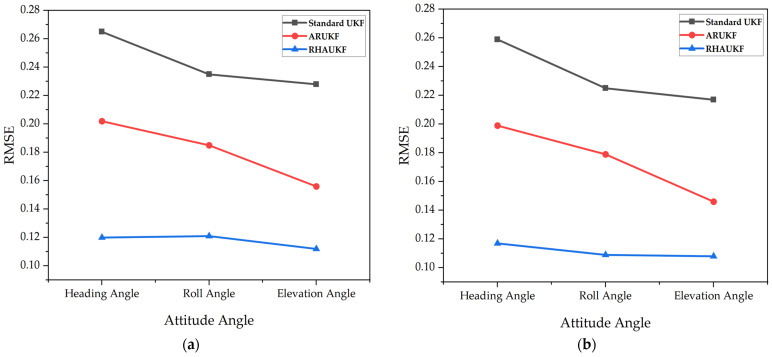
The root mean square errors of the attitude angle parameters obtained in *T*_1_ and *T*_2_: (**a**) errors in the time period *T*_1_; (**b**) errors in the time period *T*_2_.

**Figure 9:**
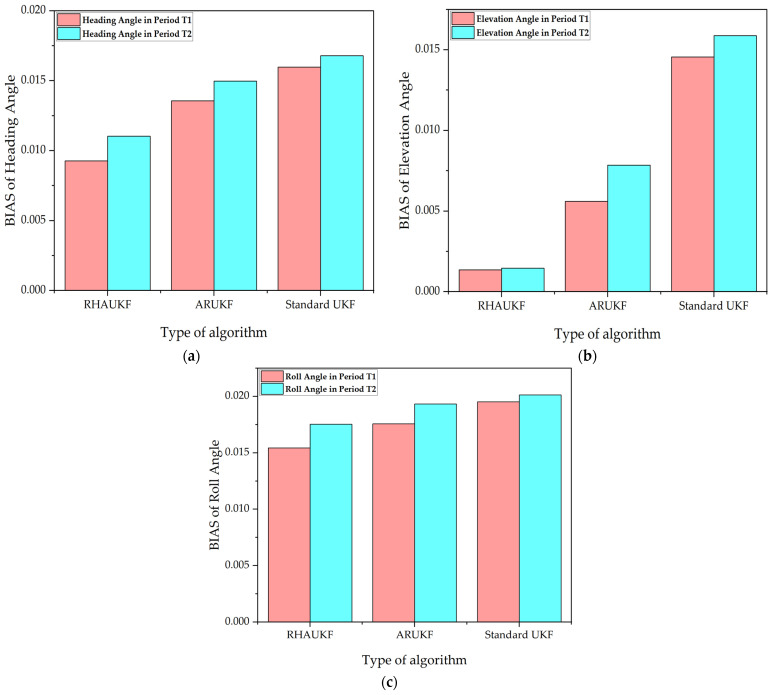
The deviations of the attitude angle parameters obtained by the three algorithms in the second case under $T_{1}$ and $T_{2}$: (**a**) BIAS of heading angle; (**b**) BIAS of elevation angle; (**c**) BIAS of roll angle.

Figure 8 shows the root mean square errors of the attitude angle parameters obtained by the three algorithms in the second case, and Figure 9 shows deviation diagrams of the attitude angle parameters.

**Figure 10:**
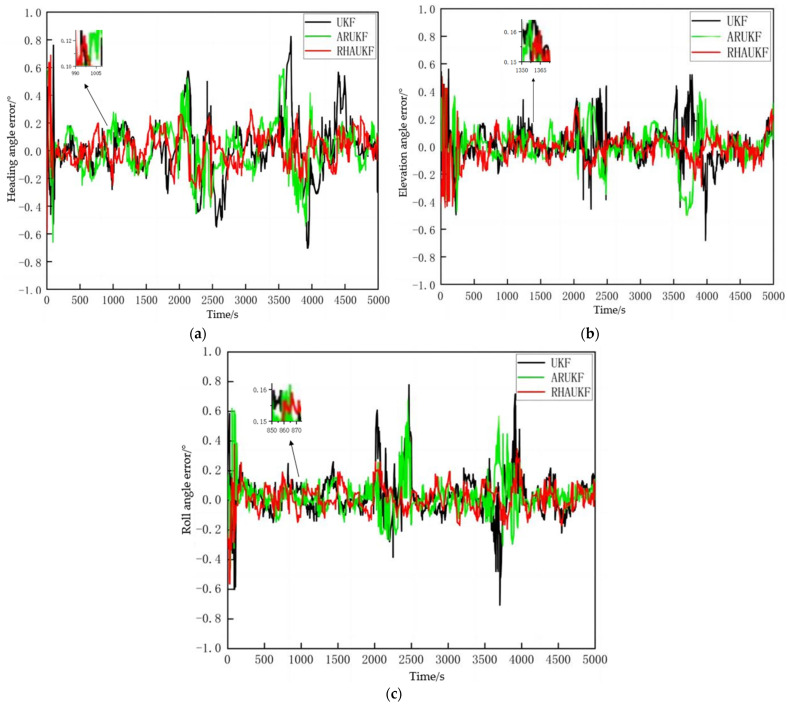
The attitude angle error curves of the three algorithms in the third case: (**a**) heading angle errors; (**b**) pitch angle errors; (**c**) roll angle errors.

Figure 10 depicts the error curves for the heading angles, pitch angles, and roll angles calculated by the three different filtering algorithms in the third case. When accurate noise statistics were available, the traditional UKF, the ARUKF, and the proposed RHAUKF exhibited comparable accuracies in estimating heading, pitch, and roll, with errors generally falling within the ranges of [−0.18°, 0.23°], [−0.14°, 0.16°], and [−0.11°, 0.15°], respectively, thereby demonstrating their effectiveness in typical underwater navigation scenarios.

During the periods $T_{1}$ and $T_{2}$, the increased measurement noise covariance resulted in degraded heading, pitch, and roll estimation accuracy for the standard UKF. Specifically, the UKF exhibited heading, pitch, and roll errors within the ranges of [−0.44°, 0.49°], [−0.38°, 0.32°], and [−0.28°, 0.48°] during $T_{1}$ and within the ranges of [−0.56°, 0.68°], [−0.62°, 0.45°], and [−0.62°, 0.61°] during $T_{2}$. While the ARUKF exhibited improved accuracy compared to the UKF, its errors remained relatively large under the increased noise conditions, specifically within the ranges of [−0.38°, 0.42°], [−0.28°, 0.22°], and [−0.21°, 0.38°] during $T_{1}$ and within the ranges of [−0.48°, 0.46°], [−0.38°, 0.28°], and [−0.21°, 0.46°] during $T_{2}$. In contrast, the proposed RHAUKF algorithm maintained significantly higher accuracy, with errors remaining within the ranges of [−0.18°, 0.19°], [−0.12°, 0.19°], and [−0.12°, 0.18°] during $T_{1}$ and within the ranges of [−0.13°, 0.19°], [−0.18°, 0.13°], and [−0.13°, 0.16°] during $T_{2}$, indicating a substantial improvement in filtering accuracy and robustness against uncertain process noise, a critical advantage for reliable underwater navigation.

**Figure 11:**
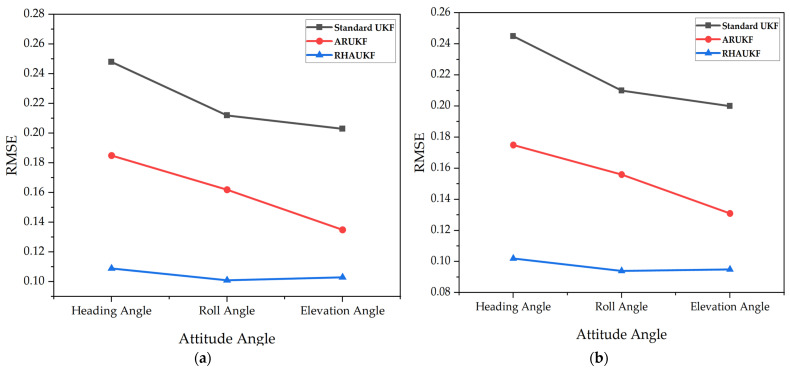
The root mean square errors of the attitude angle parameters: (**a**) errors in the $T_{1}$ time period; (**b**) errors in the $T_{2}$ time period.

**Figure 12:**
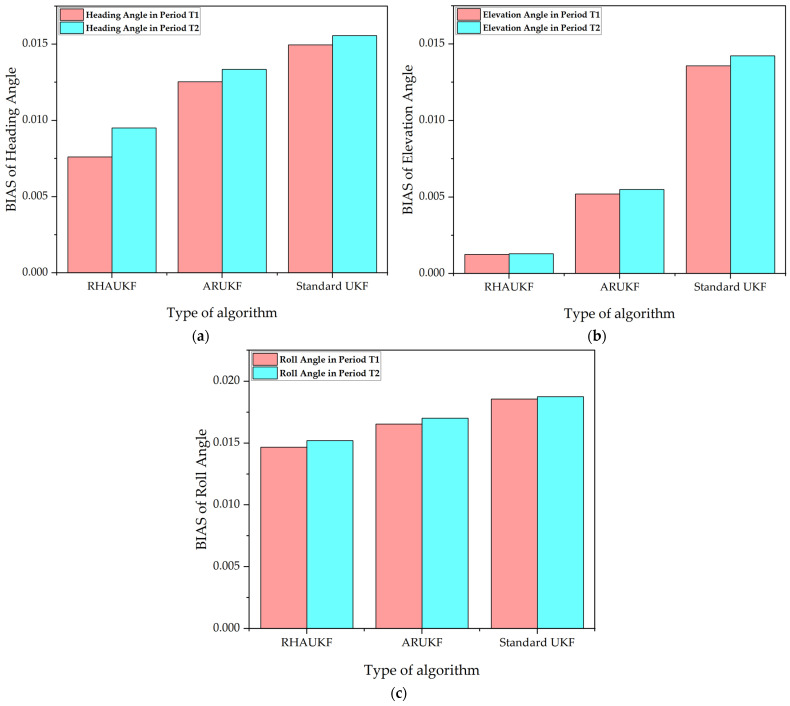
The deviations of the attitude angle parameters obtained by the three algorithms in the third case: (**a**) BIAS of heading angle, (**b**) BIAS of elevation angle, (**c**) BIAS of roll angle.

Figure 11 shows the root mean square errors of the attitude angle parameters obtained by the three algorithms in one case, and Figure 12 shows deviation diagrams of the attitude angle parameters.

The above experimental results display that the root mean square errors and deviations of the attitude angles of the RHAUKF were the smallest in the two time periods *T*_1_ and *T*_2_ and were significantly smaller than those of the classical UKF and the ARUKF. Compared with the UKF algorithm, the heading angle, pitch angle, and roll angle accuracies under the ARUKF algorithm increased by 19%, 14%, and 23% and by 14%, 20%, and 27%, respectively. The heading angle, pitch angle, and roll angle accuracies under the RHAUKF algorithm improved by 53%, 54%, and 53% and by 43%, 49%, and 53%, respectively. The RHAUKF is better than the ARUKF.

**Figure 13:**
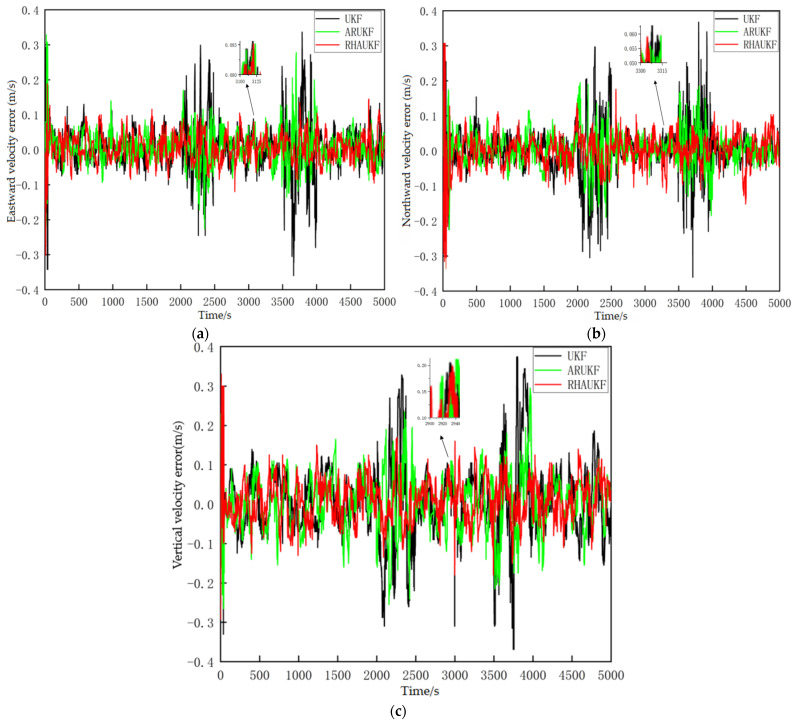
Three-axis velocity error curves for the six methods: (**a**) eastward velocity errors; (**b**) northward velocity errors; (**c**) vertical velocity errors.

As shown in Figure 13, when accurate noise statistics are known, the three algorithms exhibit comparable accuracy in estimating the three-dimensional velocity. The resulting errors in east, north, and down velocities were approximately within the ranges of [−0.11 m/s, 0.13 m/s], [−0.12 m/s, 0.10 m/s], and [−0.15 m/s, 0.17 m/s], respectively, demonstrating their effectiveness under ideal conditions typical for underwater vehicle navigation performance analysis.

During periods $T_{1}$ and $T_{2}$, where process noise variance changed, the UKF exhibited east, north, and down velocity errors approximately within the ranges of [−0.25 m/s, 0.28 m/s], [−0.29 m/s, 0.31 m/s], and [−0.28 m/s, 0.33 m/s] during $T_{1}$ and within the ranges of [−0.35 m/s, 0.35 m/s], [−0.36 m/s, 0.38 m/s], and [−0.37 m/s, 0.38 m/s] during $T_{2}$. The ARUKF, under the same conditions, yielded velocity errors within the ranges of [−0.23 m/s, 0.18 m/s], [−0.19 m/s, 0.19 m/s], and [−0.26 m/s, 0.22 m/s] during $T_{1}$ and within the ranges of [−0.18 m/s, 0.26 m/s], [−0.20 m/s, 0.19 m/s], and [−0.25 m/s, 0.30 m/s] during $T_{2}$. These results highlight the impact of varying process noise on velocity estimation accuracy for both algorithms, underscoring the importance of robust filtering techniques in challenging underwater navigation environments.

**Figure 14:**
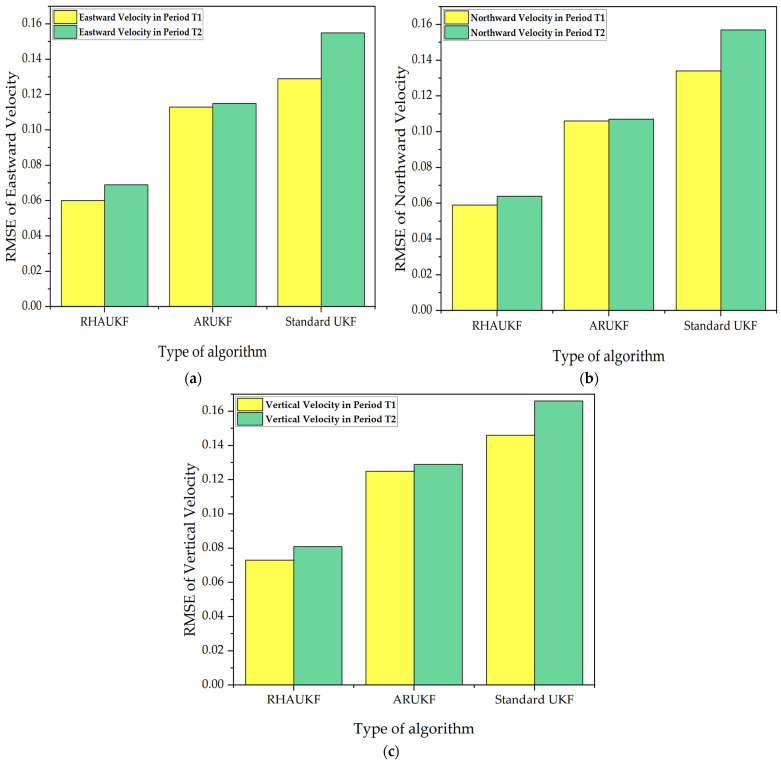
The root mean square errors of the three-way velocities: (**a**) RMSEs of eastward velocities; (**b**) RMSEs of northward velocities; (**c**) RMSEs of vertical velocities.

**Figure 15:**
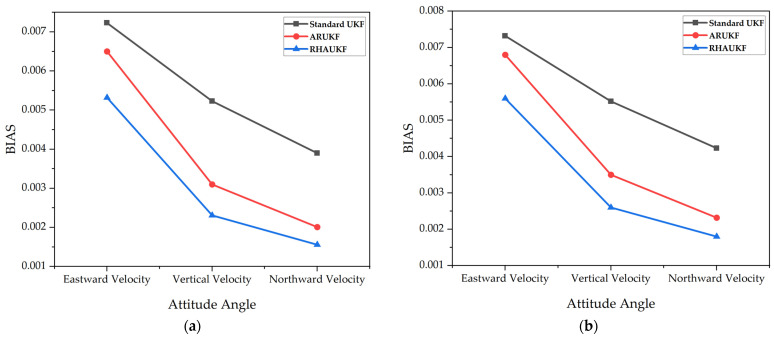
The deviations of three-way velocities: (**a**) the errors in the $T_{1}$ time period; (**b**) the errors in the $T_{2}$ time period.

For the two time periods, $T_{1}$ and $T_{2}$, the root mean square errors and deviations of the three-way velocities of the three algorithms are exposed in Figure 14 and Figure 15, respectively.

Although the results show some improvements with the ARUKF algorithm, the accuracy of velocity estimation in the period was limited, and the error was kept in a relatively wide range. In contrast, the proposed RHAUKF algorithm exhibited superior performance in terms of three-dimensional velocity error. During both $T_{1}$ and $T_{2}$, the RHAUKF achieved velocity errors (east, north, and down) comparable to the best results obtained under known noise statistics, significantly outperforming both the UKF and the ARUKF algorithms, thus showcasing its robustness and effectiveness for underwater navigation applications, where accurate velocity estimation is crucial.

The RHAUKF algorithm consistently achieved the lowest three-dimensional velocity RMSEs during both $T_{1}$ and $T_{2}$, significantly outperforming both the standard UKF and the ARUKF algorithms, as shown in Figure 14 and Figure 15. Compared to the standard UKF, the ARUKF exhibited improvements in east, north, and down velocity accuracy of 12%, 21%, and 14% during $T_{1}$ and 26%, 31%, and 22% during $T_{2}$. The proposed RHAUKF algorithm achieved substantially greater accuracy improvements of 53%, 55%, and 50% during $T_{1}$ and 55%, 59%, and 51% during $T_{2}$, surpassing even the ARUKF and highlighting its superior robustness for underwater navigation applications subject to dynamic noise conditions.

**Figure 16:**
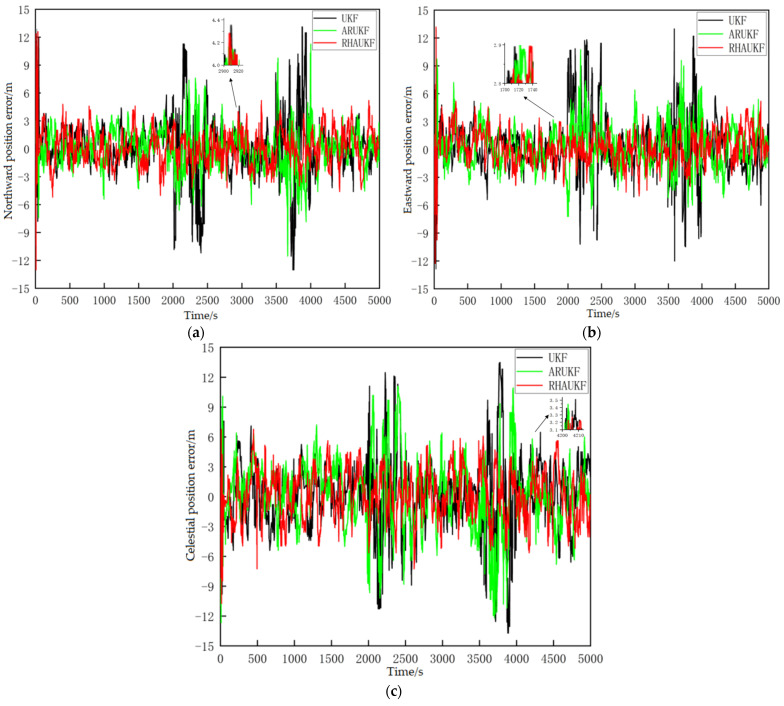
Three directional position error curves under the three algorithms: (**a**) eastward position errors; (**b**) northward position errors; (**c**) celestial position errors.

Figure 16 shows the three position error curves calculated by UKF, ARUKF and the proposed RHAUKF. When precise noise statistics were known, the traditional UKF, the ARUKF, and the proposed RHAUKF demonstrated comparable accuracy in estimating position, with resulting east, north, and down position errors approximately within the ranges of [−4.0 m, 4.5 m], [−3.5 m, 4.0 m], and [−5.5 m, 6.2 m], respectively. These results highlight the algorithms’ performance under ideal conditions, forming a benchmark for evaluating their robustness under more challenging scenarios typical of underwater navigation.

During the two time intervals of $T_{1}$ and $T_{2}$, characterized by variations in process noise variance, the UKF yielded east, north, and down position errors approximately within the ranges of [−10.5 m, 12.5 m], [−10.8 m, 11.8 m], and [−11.7 m, 12.2 m] for T_1_ and within the ranges of [−12.5 m, 13 m], [−13.5 m, 13 m], and [−11.5 m, 12.2 m] for T_2_. The ARUKF revealed slightly improved accuracy compared to the UKF, with errors within the ranges of [−7.5 m, 10.5 m], [−7.0 m, 7.0 m], and [−9.8 m, 11.0 m] during $T_{1}$ and within the ranges of [−7.0 m, 9.5 m], [−12.0 m, 11.0 m], and [−12.0 m, 10.5 m] during $T_{2}$. However, the proposed RHAUKF algorithm demonstrated the highest position estimation accuracy, achieving errors comparable to those obtained under known noise statistics during both periods. This represents a significant improvement in filtering accuracy compared to both the UKF and the ARUKF, highlighting its robustness for underwater navigation in the presence of dynamic noise.

**Figure 17:**
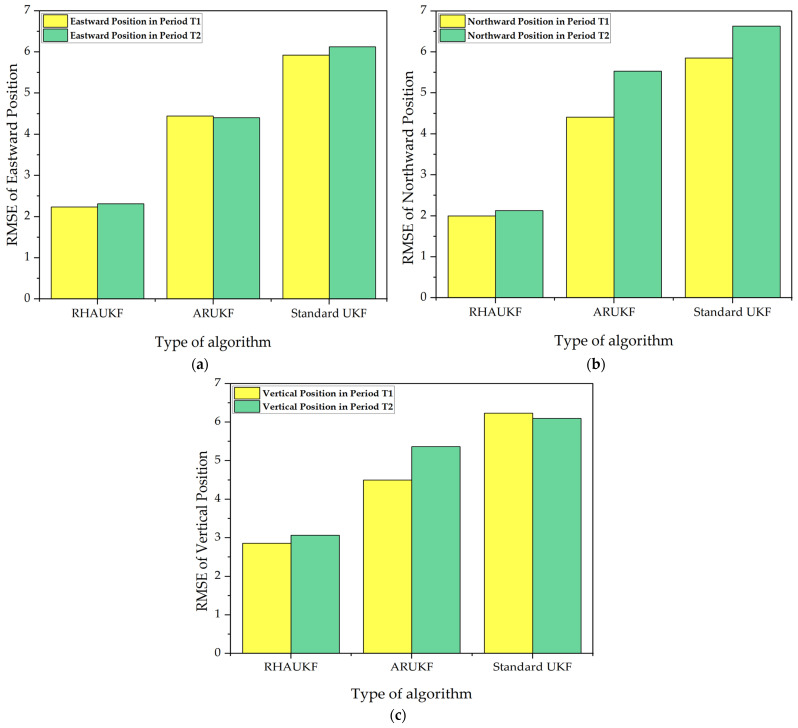
The root mean square errors of the three-way positions: (**a**) RMSEs of eastward position; (**b**) RMSEs of northward position; (**c**) RMSEs of vertical position.

**Figure 18:**
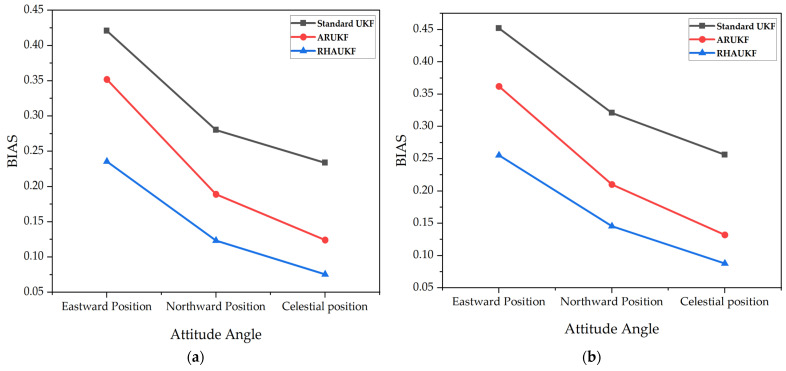
Deviations of three-way positions: (**a**) deviation curves during the period $T_{1}$; (**b**) deviation curves during the period $T_{2}$.

The root mean square errors and deviations of the three-way positions of the three algorithms in the two time periods, $T_{1}$ and $T_{2}$, are shown in Figure 17 and Figure 18, below.

Figure 17 and Figure 18 show that the RHAUKF algorithm consistently achieved the lowest three-dimensional position RMSEs during both periods, $T_{1}$ and $T_{2}$, significantly outperforming both the standard UKF and the ARUKF. Specifically, during $T_{1}$, the ARUKF improved east, north, and down position accuracies by 25%, 25%, and 28%, respectively, compared to the UKF; and during $T_{2}$, these improvements were 28%, 16%, and 12%. The RHAUKF algorithm yielded even more substantial improvements, with accuracy gains of 62%, 65%, and 54% in $T_{1}$ and 62%, 68%, and 50% in $T_{2}$, clearly surpassing the ARUKF and demonstrating its superior performance for robust underwater navigation.

The simulation results demonstrate that, under ideal conditions with precisely known system noise, the designed SINS/DVL integrated navigation system achieves high navigation accuracy, enabling precise positioning. In such cases, the traditional UKF, the ARUKF, and the proposed RHAUKF perform comparably. However, when prior noise statistics are unknown or inaccurate—a more realistic scenario for underwater navigation—the proposed adaptive UKF algorithm achieves significantly enhanced filtering performance compared to the UKF and the ARUKF. This adaptive algorithm effectively suppresses noise interference, resulting in system errors that closely approximate those achieved with precisely known noise characteristics, highlighting its robustness for real-world underwater applications.

## 5. Conclusions

This paper presents a novel adaptive Unscented Kalman Filter (RHAUKF) specially designed for SINS/DVL integrated navigation systems, which leverages a maximum likelihood criterion in conjunction with a receding-horizon estimation technique. Compared to the traditional UKF algorithm, the ARUKF algorithm shows a 20% improvement in angular accuracy, a 25% enhancement in three-way velocity, and a 28% boost in three- dimensional positioning. However, it still grapples with issues of low filtering accuracy or even divergence. In contrast, the RHAUKF builds a noise estimation model grounded in the maximum likelihood principle. This model is further refined through receding-horizon estimation and employs a Newton–Raphson method for efficient computation, resulting in a significantly more accurate and stable algorithm. Extensive simulations underscore the RHAUKF’s superior filtering accuracy and stability, highlighting its capability to effectively counteract divergence under the challenging conditions often found in underwater navigation environments. The algorithm proposed in this paper goes beyond enhancing anti-noise capabilities; it also contributes to more stable underwater navigation for UUVs and can aid in predicting complex surrounding environments. Looking ahead, we hope to contribute to the advancement of unmanned underwater vehicles.

## Figures and Tables

**Table 1:** Abbreviations, symbols and their corresponding meanings used in this article.

Abbreviations and Symbols	Definition
x	The input variable
$\bar{x}$	The mean value
$P_{xx}$	The variance
$\left\{x_{i}\right\}$	The sigma point set of the input variable
$X_{k}$ and $Z_{k}$	The system state component and the measurement component at time k
$W_{k}$ and $V_{k}$	The system state noise and the measurement noise
f(∙) and h(∙)	The nonlinear system state function and the measurement function
$W_{k}$ and $V_{k}$	Uncorrelated Gaussian white noise
$q_{k}$ and $r_{k}$	The covariance matrices of $W_{k}$ and $V_{k}$
$Q_{k}$	A non-negative definite matrix
$R_{k}$	A positive definite matrix
$p\left(Z_{1:k}|\theta \right)$	The joint probability density function
$\varepsilon_{k}$	Zero-mean Gaussian white noise
$H_{k}$	The measurement matrix
$v^{n}$	The simulation experiment
M	The time period of the simulation experiment
$E\left[\cdot \right]$	The sign of the expectation (i.e., the mean)
$\hat{f}\left(x\right)$	The model’s prediction of a given input
$f\left(x\right)$	The real function value

**Table 2:** Summary of the literature review.

Author	Name of Algorithm	Merits and Demerits
He K	An asynchronous adaptive direct Kalman filter algorithm	Improves navigation accuracy by adaptively adjusting the measurement noise variance matrix
Luo	A robust Kalman filter	Eliminates process uncertainties and measurement anomalies caused by severe maneuvering
Lu Zhang	Square root information filter	Improves the convergence rate of the initial alignment of a strapdown inertial navigation system
He Shan	An improved strong tracking quadrature Kalman filter algorithm	Introduces the idea of a strong tracking filtering algorithm into the nonlinear filtering algorithm
Ye Chen	A strong tracking UKF algorithm	An algorithm with higher accuracy and stronger anti-noise ability
Chen Xiaofeng	A tracking Unscented Kalman Filter algorithm	The computational power is more complex
Chen Guangwu	An integrated navigation algorithm	Improves the performance of state estimation
S Guo	A federated Kalman Filter	Reduces the pollution to other sub-filters caused by feedback and reduces the abnormal positioning caused by faults
González	The improved Kalman filter	Improves the navigation accuracy of underwater AUVs in deep-sea areas
Gao	An adaptive PD control algorithm	Has good stability and adaptability

## Data Availability

Data are contained within the article.

## References

[1] Sahoo A., Dwivedy S.K., Robi P.S. (2019). Advancements in the field of autonomous underwater vehicle. Ocean Eng..

[2] Merveille FF R., Jia B., Xu Z., Fred B. (2024). Enhancing Underwater SLAM Navigation and Perception: A Comprehensive Review of Deep Learning Integration. Sensors.

[3] Jin F., Cheng B., Luo W. (2024). Data-Driven Based Path Planning of Underwater Vehicles Under Local Flow Field. J. Mar. Sci. Eng..

[4] He L., Zhang Y., Li S., Li B., Yuan Z. (2024). Three-Dimensional Path Following Control for Underactuated AUV Based on Ocean Current Observer. Drones.

[5] Liu Y., He L., Fan G., Wang X., Zhang Y. (2024). A Co-Localization Algorithm for Underwater Moving Targets with an Unknown Constant Signal Propagation Speed and Platform Errors. Sensors.

[6] Zhu T., Li J., Duan K., Sun S. (2024). Study on the Robust Filter Method of SINS/DVL Integrated Navigation Systems in a Complex Underwater Environment. Sensors.

[7] Li J., Gu M., Zhu T., Wang Z., Zhang Z., Han G. (2023). Research on error correction technology in underwater SINS/DVL integrated positioning and navigation. Sensors.

[8] Li P., Liu Y., Yan T., Yang S., Li R. (2023). A robust INS/USBL/DVL integrated navigation algorithm using graph optimization. Sensors.

[9] Zhang Y., Zhang Y., Yu J., Zhao F., Zhu S. (2024). Structural Online Damage Identification and Dynamic Reliability Prediction Method Based on Unscented Kalman Filter. Sensors.

[10] Wang Y., Xie C., Liu Y., Zhu J., Qin J. (2024). A Multi-Sensor Fusion Underwater Localization Method Based on Unscented Kalman Filter on Manifolds. Sensors.

[11] Wang J., Xia L., Peng L., Li H., Cui Y. (2023). Efficient uncertainty propagation in model-based reinforcement learning unmanned surface vehicle using unscented kalman filter. Drones.

[12] Yao Y., Xu X., Hou L., Deng K., Xu X. (2020). A simple and precise correction method for DVL measurements under the dynamic environment. IEEE Trans. Veh. Technol..

[13] He K., Liu H., Wang Z. (2020). A novel adaptive two-stage information filter approach for deep-sea USBL/DVL integrated navigation. Sensors.

[14] Tavares A.J.A., Oliveira N.M.F. (2024). A Novel Approach for Kalman Filter Tuning for Direct and Indirect Inertial Navigation System/Global Navigation Satellite System Integration. Sensors.

[15] Liu H., Gong Z., Shen J., Li Y., Long Q. (2024). A Method for Measuring the Error Rules in Visual Inertial Odometry Based on Scene Matching Corrections. Micromachines.

[16] He S., Zhao X., Shi X. (2018). Improved strong tracking quadrature Kalman filter algorithm. Comput. Technol. Dev..

[17] Ye C., Cui S. (2018). A strong tracking UKF and its application in GPS/SINS deep integrated navigation. Missile Aerosp. Deliv. Technol..

[18] Chen G., Wang S., Si Y., Zhou X. (2024). Research on integrated navigation algorithm based on adaptive interacting multiple Kalman filter model. J. Electron. Inform..

[19] Chang C.W., Lo L.Y., Cheung H.C., Feng Y., Yang A.S., Wen C.Y., Zhou W. (2022). Proactive guidance for accurate UAV landing on a dynamic platform: A visual–inertial approach. Sensors.

[20] Chen X., Zhuang W., Li S., Wu C., Fan J., Liu D. (2024). An integrated navigation method based on strong tracking unscented Kalman filter algorithm. Electromechanical Eng. Technol..

[21] Li J. (2023). Research On Fault-Tolerant Multi-Sensor Integrated Navigation Algorithm Based on Federated Kalman Filter. Master’s Thesis.

[22] Bao L.-H., Zeng Q.-J., Zhu Z.-Y., Dai X.-Q., Zhao Q. (2019). AUV Docking Recovery Based on USBL Integrated Navigation Method. Proceedings of the 2019 Chinese Automation Congress (CAC).

[23] Bao J., Li D., Qiao X., Rauschenbach T. (2020). Integrated navigation for autonomous underwater vehicles in aquaculture: A review. Inf. Process. Agric..

[24] Wang B., Huang L., Liu J., Deng Z., Fu M. (2020). A support vector regression-based integrated navigation ethod for underwater vehicles. IEEE Sens. J..

[25] Wang C., Wu H., Wang F., Cheng L. (2008). Trajectory tracking control of mobile robot based on Backstepping. Mod. Electron. Technol..

[26] Chen Z., Wang H., Bian X.-Q., Jia H. (2013). Inverse Depth Control by AUV Stabilized Neural Networks Based on Feedback Gain. J. Control. Decis..

[27] Gao Z. (2004). Adaptive PD Control Algorithm for Vehicle Orientation Preview. Chin. J. Mech. Eng..

[28] Daid A., Busvelle E., Aidene M. (2021). On the convergence of the unscented Kalman filter. Eur. J. Control.

[29] Ullah I., Shen Y., Su X., Esposito C., Choi C. (2019). A localization based on unscented Kalman filter and particle filter localization algorithms. IEEE Access.

[30] Brossard M., Bonnabel S., Condomines J.P. (2017). Unscented Kalman filtering on Lie groups. Proceedings of the 2017 IEEE/RSJ International Conference on Intelligent Robots and Systems (IROS).

[31] Xu Y. (2020). A Moving Target Tracking Algorithm Based on Unscented Kalman Filter. Firepower Command. Control..

[32] Liu Y., Wen D., Yi H., Yin Q. (2024). Unscented Kalman Filter Estimation of Spatial Target Feature Information. Sci. Technol. Eng..

[33] Deng B., Liu M. (2024). Design of Inertial Navigation System for a Small Underwater Vehicle. Comput. Digit. Eng..

